# Patterns of Organ‐Specific Proteomic Aging in Relation to Lifestyle, Diseases, and Mortality

**DOI:** 10.1111/acel.70251

**Published:** 2025-10-08

**Authors:** Qi Wang, Jingting Huang, Qida He, Mengtong Sun, Michael P. Snyder, Linyan Li

**Affiliations:** ^1^ Department of Data Science, College of Computing City University of Hong Kong Hong Kong Hong Kong SAR China; ^2^ Department of Infectious Diseases and Public Health, Jockey Club College of Veterinary Medicine and Life Sciences City University of Hong Kong Hong Kong Hong Kong SAR China; ^3^ Department of Genetics, School of Medicine Stanford University Stanford California USA; ^4^ Center for Genomics and Personalized Medicine Stanford University Stanford California USA

**Keywords:** aging, chronic disease, disease incidence, lifestyle, longevity, proteomics

## Abstract

Aging occurs in a heterogeneous manner across different organs, leading to varying risks of chronic diseases and mortality. Biological age offers a more comprehensive reflection of the aging process and is a stronger predictor of disease risk and lifespan. Recent advances in plasma proteomics have enabled the development of organ‐specific aging clocks, revealing the distinct aging trajectories and their clinical implications. We used protein‐based aging estimators for 11 organs, applying them to plasma data using elastic net regularization. A comprehensive analysis of associations was conducted with 86 lifestyle and environmental factors, 657 diseases through phenome‐wide association studies (PheWAS), and all‐cause mortality. Our findings revealed that organ aging is influenced by lifestyle factors and baseline health conditions, highlighting its dynamic and modifiable nature. Additionally, accelerated organ aging is associated with a higher incidence of disease and an increased risk of all‐cause mortality, particularly when it occurs earlier in life. Our large‐scale lifestyle atlas and PheWAS offer actionable insights into the modifiable drivers of organ aging, advancing strategies for disease prevention and longevity.

## Graphical abstract

We estimated 11 organ‐specific proteomic aging using the elastic net regularization model. Our findings revealed that organ‐specific proteomic aging is influenced by lifestyle factors and baseline health conditions, and can further predict all‐cause mortality and the onset of various diseases.

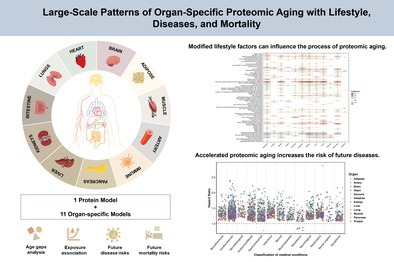

## 1 Introduction

Aging is the primary risk factor for progressive physiological decline across organ systems, driving susceptibility to age‐related diseases and mortality (Mutz et al. 2021; Niccoli and Partridge 2012; Partridge et al. 2018). However, this process varies significantly among individuals, even those of the same chronological age (Elliott et al. 2021). Such variability arises from differences in biological aging, which might be shaped by factors such as overall health and lifestyle choices (Guo et al. 2022; Thomas et al. 2023; Wu et al. 2024). Unlike chronological age, biological aging reflects cumulative molecular and cellular damage that disrupts organ function, making it a more robust predictor of disease risk and mortality (Ferrucci et al. 2020; Khan et al. 2017; Moqri et al. 2023). This distinction underscores the need for accurate biological aging measures at the systemic and organ‐specific levels (Ahadi et al. 2020; Belsky et al. 2015; Lu et al. 2020; Mutz et al. 2024; Palliyaguru et al. 2021).

Critically, aging does not progress uniformly across organs within the same individual (Nie et al. 2022; Tian et al. 2023; Tuttle et al. 2020). Animal studies have identified distinct molecular aging signatures across organs (Hajat and Stein 2018; Schaum et al. 2020; Tabula Muris 2020; Zahn et al. 2007), and recent human research revealed similar variability in rates of organ and biological process aging, highlighting the importance of assessing aging at the individual organ level (Oh et al. 2025; Rando and Wyss‐Coray 2021). Traditional approaches rely on clinical indicators to assess aspects of organ function, such as blood biochemical parameters, MRI imaging of the brain, epigenetic markers, and concentrations of proteins specific to certain organs in the bloodstream (Levine et al. 2018; Putin et al. 2016; Tian et al. 2023). They often lack the specificity for precise organ‐level aging assessments (Oh et al. 2023).

Recent advances in plasma proteomics have led to the development of organ‐specific aging clocks, revealing distinct aging trajectories and their clinical relevance (Coenen et al. 2023; Lehallier et al. 2019; Macdonald‐Dunlop et al. 2022; Rutledge et al. 2022; Sayed et al. 2021; Sun et al. 2023; Tanaka et al. 2020). Pioneering work by Oh et al. (2023) introduced organ‐specific proteomic clocks using Gene Tissue Expression Atlas (GTEx)‐derived organ‐enriched proteins and least absolute shrinkage and selection operator (lasso) models applied to plasma data, linking them to health outcomes. Some studies have further developed a “proteomic age clock” based on plasma proteomic expression profiles across diverse populations, suggesting associations with multimorbidity and all‐cause mortality risk (Argentieri et al. 2024). However, the extensive effects of modifiable lifestyle factors, disease associations, and mortality risks remain insufficiently explored. A comprehensive analysis is necessary to understand the modifiable drivers and health consequences of proteomic aging across organs.

In this study, we applied organ‐specific proteomic age estimators derived from plasma protein data from the UK Biobank (UKB). Through a comprehensive phenome‐wide association study (PheWAS) analysis of 86 lifestyle factors and 657 diseases, we assessed their impact on disease risks and all‐cause mortality. Our mapping of organ‐resolved aging patterns provided a nuanced understanding of biological aging beyond traditional whole‐body measures. Additionally, we identified key modifiable factors that decelerate organ decline, offering actionable insights for interventions. Our findings contribute to the growing body of knowledge regarding the lifestyle factors and pathways influencing organ aging, with the potential to improve health span and reduce disease risk in aging populations.

## 2 Materials and Methods

### 2.1 Study Participants and Identification of Organ‐Specific Plasma Proteins

The UKB is a population‐based prospective cohort study of over 500,000 individuals aged 37 to 73 years at recruitment between 2006 and 2010. Detailed information on available phenotypic data is accessible at https://biobank.ndph.ox.ac.uk/showcase/. We primarily included 53,013 participants randomly selected from the UKB cohort with available baseline Olink Explore proteomic data in this study. Data processing and quality control procedures followed established protocols described in the previous study (Sun et al. 2023).

A total of 2923 plasma proteins were primarily included in our analysis. We performed additional quality control steps in the following order. First, 8063 participants with more than 49.9% protein missing values were removed, followed by removing 7 proteins with missing values in more than 10% of samples. Lastly, 340 samples with discordant reported sex and genetic sex were removed. This resulted in a post‐quality control dataset consisting of 44,610 participants with 2916 proteins for further analysis. To identify organ‐specific plasma proteins, we employed the methodology described in previous studies (Oh et al. 2023). Organ‐enriched genes were identified as exhibiting at least a fourfold higher expression level in one organ compared to all others, derived from human tissue bulk RNA‐seq data provided by the GTEx. These organ‐enriched genes were then mapped to their corresponding proteins, enabling the identification of organ‐specific plasma proteins within the UKB data (Tables S1 and S2, Figure S1).

### 2.2 Organ‐Specific Proteomic Age Estimation

To prepare the proteomics data for model training, the *k*‐nearest neighbors (*k*‐NN) algorithm was employed to impute the missing values (Hastie et al. 2024). We trained a *k*‐nearest neighbors model via the impute.knn function from the *impute* R package, with the number of neighbors (*k*) set to the square root of the sample size (*k* = 211). Protein values were normalized based on the means and standard deviations of proteins within gender groups before model training. Chronological age at baseline was calculated for each participant using birth year, birth month, and the dates of initial assessments. Chronological age was computed as the interval (in months) between birth and assessment dates.

We employed elastic net regularization, which effectively combines the strengths of lasso and ridge regression (Watanabe et al. 2023). This method enables simultaneous feature selection and coefficient shrinkage by incorporating both L1 and L2 penalties. Elastic net regression models were trained separately for males and females to predict organ‐specific ages. Data was randomly split into training (80%) and test (20%) sets. A hyperparameter grid was defined, with alpha (controlling the mix of L1 and L2 regularization) ranging from 0 to 1 in increments of 0.05 and lambda (regularization strength) spanning a logarithmic scale from 1 × 10^−2^ to 1. Hyperparameter tuning was performed using a repeated 5‐fold cross‐validation strategy, optimizing for mean absolute error (MAE). The train function from the *caret* R package (Kuhn 2008) was used to conduct a grid search over the hyperparameter space. The best‐performing alpha and lambda values were selected, and a final elastic net model was trained on the entire training dataset using these optimal hyperparameters. We then applied the trained model to both training and test sets to generate proteomic age estimations. Organ‐specific plasma proteins were used to train individual aging models for each organ. Additionally, a “Protein” aging model was trained using all available proteins.

Age gap was defined as the difference between predicted age and chronological age, with positive values indicating older predicted age and negative values indicating younger predicted age relative to chronological age. To correct systematic biases in age prediction (i.e., the tendency to underestimate in older individuals and overestimate in younger ones), we performed a linear regression of predicted age (PredAge) on chronological age. The resulting slope (α) and intercept (β) were used to adjust predictions using the following formula (de Lange and Cole 2020):

$$\text{PredAge}\;\left(\mathrm{age}\;\text{bias adjusted}\right)=\text{PredAge}+\left[\mathrm{Age}-\left(\alpha \times \mathrm{Age}+\beta \right)\right]$$

After bias correction, the age gap was derived by subtracting chronological age from the adjusted predicted age, followed by *z*‐score normalization within each aging model. Individuals classified as extreme agers had a *z*‐scored age gap exceeding 1 or below −1 in any aging model.

### 2.3 Lifestyle Factors, Health Outcome, and Mortality

We tested the associations between *z*‐scored age gaps and lifestyle factors. A total of 86 variables (Table S3) were selected to capture individual differences in sociodemographic factors (e.g., education, job status, Townsend deprivation index) and lifestyle factors (e.g., diet, physical activity, and sleep). Continuous variables were *z*‐scored to have a mean of 0 and a standard deviation of 1. Linear regressions were used to evaluate the relationships between organ‐specific *z*‐scored age gaps and lifestyle factors while adjusting for sex and chronological age. Additionally, prospective associations between organ‐specific age and all‐cause mortality were examined. Mortality data were obtained via linkage to national death registries, including the NHS Central Register (Scotland) and NHS Digital (England and Wales).

In addition, we investigated the associations between *z*‐scored age gaps and incident diseases through a PheWAS approach. We used Cox proportional hazards models treating the *z*‐scored organ‐specific age gap as the exposure of interest, adjusted for chronological age and sex. Disease outcomes were defined using the Phecode Map X (https://phewascatalog.org/), which includes 3612 disease definitions organized into clinically relevant categories based on standardized ICD‐10 codes. Given our interest in incident disease and the age distribution of the sample, we excluded Phecodes corresponding to pregnancy‐related conditions, congenital anomalies, genitourinary, neonatal diseases, and sex‐specific diseases. Diagnostic codes were obtained from national hospital data and outpatient general practitioner visit data, accessed through electronic health records. To ensure model stability, we restricted our analysis to diseases with at least 100 incident events, resulting in 657 diseases in total for further analysis.

We also explored associations between organ‐specific age and 16 chronic diseases, including Parkinsonism, multiple sclerosis, stroke, dementia, depression, bipolar disorder, schizophrenia, ischemic heart disease (IHD), hypertensive diseases, chronic obstructive pulmonary disease (COPD), chronic kidney disease (CKD), diabetes, cirrhosis, osteoarthritis, osteoporosis, and cancer. All disease categories were inclusively defined, capturing the full spectrum of causes and subtypes. Further details can be found in a previous study (Aman 2023). Table S4 lists the diagnostic codes for each of the 16 chronic diseases.

### 2.4 Statistical Analysis

We conducted all statistical analyses using R (v.4.4.1). Associations between *z*‐scored age gaps and lifestyle/environmental factors were tested using linear regression. We used two‐sided t‐tests to determine whether the mean *z*‐scored age gap in specific chronic diseases was significantly different from 0. Survival probabilities based on the protein age gap were estimated using the Kaplan–Meier method (Bland and Altman 1998). Cox proportional hazards models were employed to test associations between the *z*‐scored age gap and incident outcomes (mortality and diseases). Survival outcomes were defined based on follow‐up time to the first incident event and a binary indicator denoting disease occurrence. Individuals with prevalent cases were excluded from all incident disease analyses. All models were adjusted for chronological age and sex. The Bonferroni method corrected *p*‐values for multiple comparisons via controlling the family‐wise error rate (FWER) (Bland and Altman 1995).

## 3 Results

### 3.1 Organ‐Specific Proteomic Age Estimates

**FIGURE 1:**
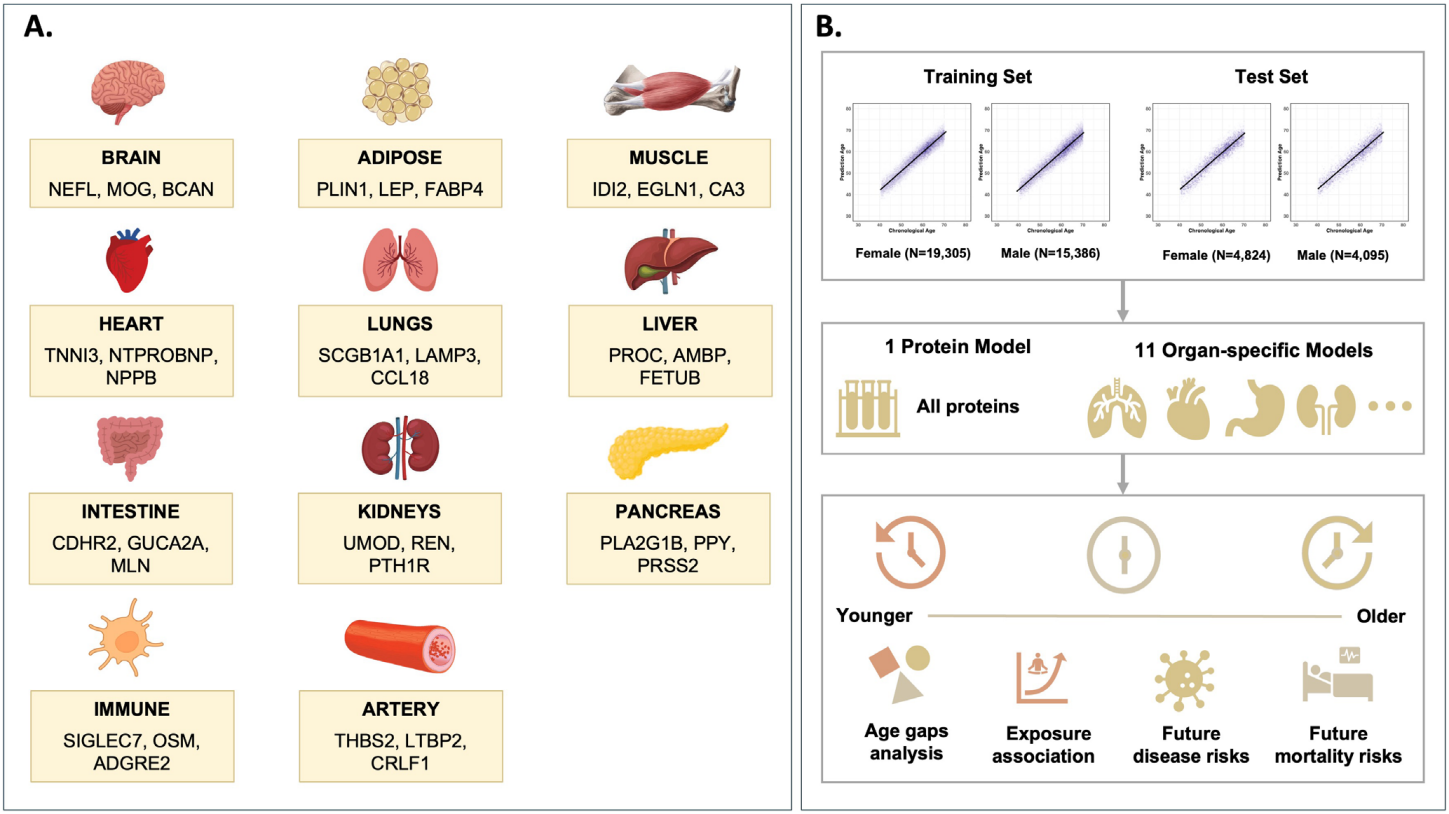
Study overview. (A) Organ systems and corresponding plasma proteins used to build normative proteomic age models. Key representative proteins are shown for each organ system. (B) Study design for estimating organ‐specific proteomic age and further analysis. Organ‐specific proteomic aging clocks were trained separately for each gender. Age gaps were characterized and tested for associations with modifiable lifestyle factors, disease risk, and mortality risk.

An overview of our study design and main analytical approaches is presented in Figure 1. We utilized plasma protein data from a subset of 44,610 UKB participants (54.09% female, aged 39–71 years) to estimate overall and organ‐specific proteomic age. Our analysis focused on 11 major organs (Aman 2023): adipose, artery, brain, heart, immune, intestine, kidney, liver, lung, muscle, and pancreas. Plasma proteins likely originating from these organs were identified and used for training the prediction model (Figure 1A, Tables S1 and S2).

Separate predictive models for proteomic age estimation were developed for female and male participants to better capture the respective protein change signatures, and top‐ranked proteins based on the absolute coefficient estimates for each prediction model were illustrated in Figure S2. Within each gender group, the dataset was randomly split into an 80% training set (Female: *N* = 19,305, Male: *N* = 16,368) and a 20% test set (Female: *N* = 4824, Male: *N* = 4113) (Figure 1B). We employed the elastic net regularization to train the aging models. We constructed a “Protein” age model using all available proteins in the post‐quality control dataset and 11 organ‐specific proteomic age models based on plasma proteins associated with each organ (Sun et al. 2023). We analyzed the characteristics of age gaps (the deviation between the predicted age and the chronological age) and investigated the associations between proteomic age and 86 lifestyle and environmental factors, as well as 657 diseases. Additionally, we assessed the impact of organ‐specific proteomic age on the risk of 16 major chronic diseases and all‐cause mortality (Figure 1B).

### 3.2 Aging Models' Performance

**FIGURE 2:**
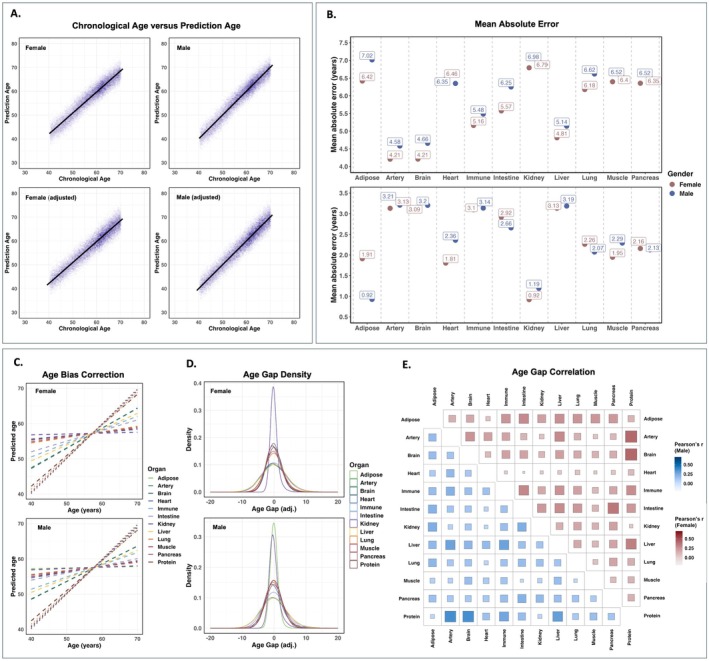
Prediction performance and bias correction in aging models. (A) Comparison of predicted age and chronological age for female and male subjects before and after bias correction. (B) MAE across different organs for males and females, before and after bias correction. (C) Age bias correction across organs for males and females. (D) Age gap density for different organs, separated by gender. (E) Correlation of age gaps across different organs for males and females.

The predictive performance of the aging models was stable across the training and test sets for both males and females, with similar MAE (Figure S3). Specifically, the MAE in the “Protein” model is 2.01 years for the training set and 2.17 years for the test set for females, and the MAE is 2.11 years for the training set and 2.33 years for the test set for males, respectively. Predictive models were also trained to estimate the proteomic age with sex as a covariate. The MAE for both‐sexes models was relatively larger than the female‐only models, and slightly lower than the male‐only models (Figure S3). Pearson's r between predicted proteomic age derived from sex‐stratified models and both‐sexes model was high (*r* = 0.99, Figure S4). We observed that all models exhibited a consistent pattern of bias, overestimating the age of younger participants while underestimating the age of older participants (Figures 2A and S5). We applied a regression of predicted age on chronological age to correct this (see methods). This adjustment significantly improved prediction accuracy, reducing MAE across all organs, ranging from 0.92 to 3.21 years, demonstrating an overall enhancement in model performance (Figure 2B,C).

### 3.3 Pairwise Correlation of Organ Age Gaps

Following bias correction, most individuals' age‐adjusted proteomic age was similar to their chronological age, with the difference centered around zero. The distribution was more concentrated in females than in males, suggesting possible sex‐related variations in proteomic aging patterns (Figure 2D). Further examination of age gap correlations across different organs revealed substantial variability, ranging from weak to strong correlations, suggesting that proteomic aging acceleration differs across organ systems (Figure 2E). High correlations among organ systems suggest they share a similar aging process, whereas low correlations indicate inconsistency in the aging process. For instance, there is a notable correlation between artery and liver age gaps and between immune and adipose age gaps. Conversely, the correlation between the heart and other organ systems' age gaps appears to be lower. To enable direct comparisons between organs in subsequent analyses, we standardized age gaps by calculating the difference between predicted and chronological age and then applying *z*‐score normalization for each aging model. In each aging model, individuals with a *z*‐scored age gap greater than 1 (defined as the “Older” group) or less than −1 (defined as the “Younger” group) were considered extreme agers. Individuals with a *z*‐scored age gap between −1 and 1 were considered a “Middle” group.

### 3.4 Organ‐Specific Age Is Influenced by Lifestyle/Environmental Factors and Baseline Health Conditions

**FIGURE 3:**
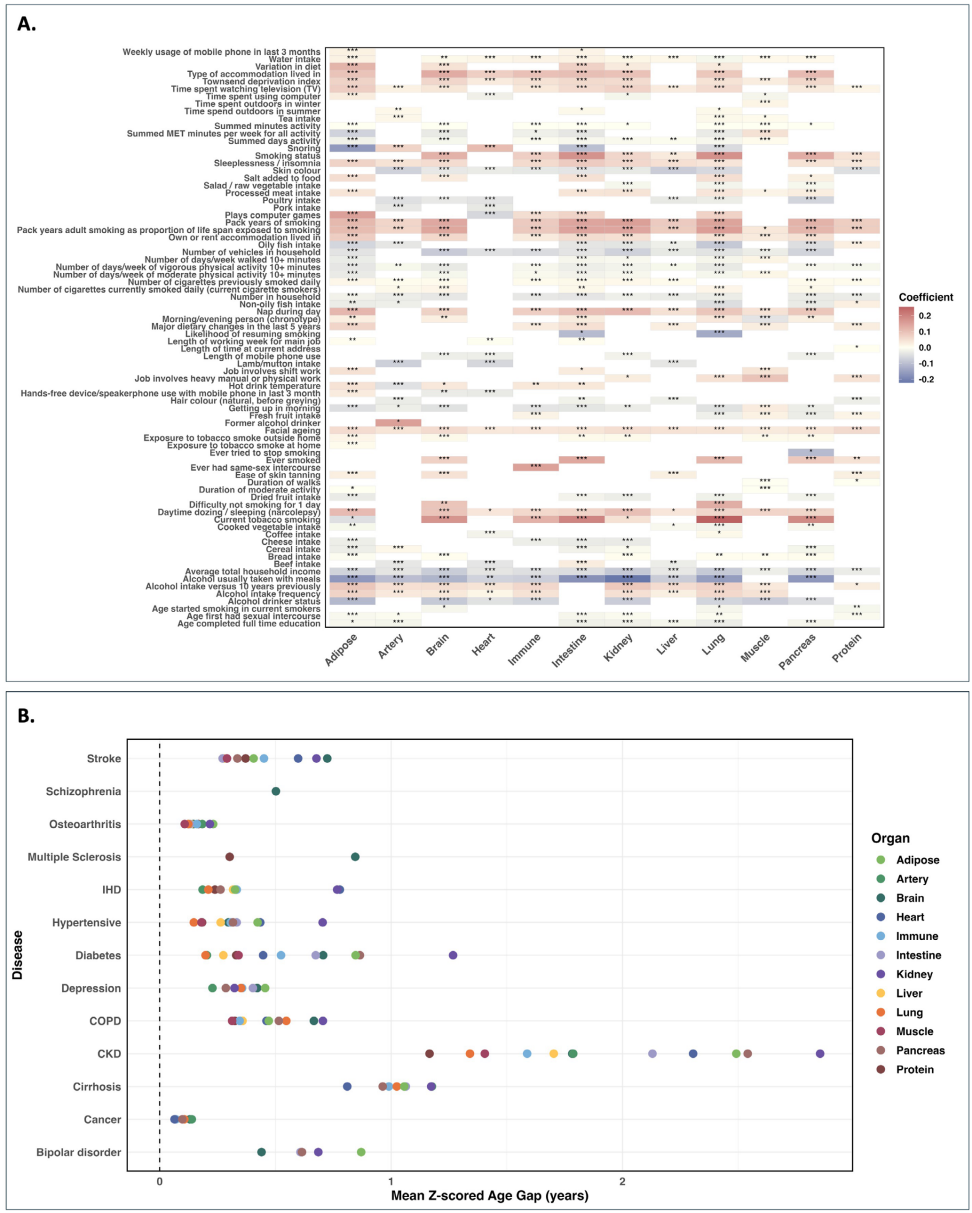
Associations between lifestyle/environmental factors, chronic disease, and organ‐specific aging. (A) Associations between 86 lifestyle and environmental factors and the *z*‐scored age gaps of 12 proteomic aging clocks. All associations were tested using linear regression and were adjusted for chronological age and sex. Coefficient estimates for the association between lifestyle/environmental factors and each *z*‐scored age gap. Bonferroni correction was used to control the family‐wise error rate (FWER) across 12 × 86 = 1032 tests. Lifestyle/environmental factors are significantly (*p* < 0.05/1032) associated with at least one organ and are shown in the figure. Significance levels are denoted as follows: “***” indicates *p.correct* < 0.001, “**” indicates 0.001 ≤ *p.correct* < 0.01, “*” indicates 0.01 ≤ *p.correct* < 0.05, and no asterisk indicates *p.correct* ≥ 0.05. (B) Mean *z*‐scored organ age gaps for individuals affected by 16 chronic diseases. Bonferroni correction was used to control the FWER across 12 × 16 = 192 tests. Mean *z*‐scored age gaps significantly (*p* < 0.05/192) different from 0 are shown in the figure.

To investigate how lifestyle and environmental factors impact proteomic aging across different organ systems, we tested the relationships between 86 lifestyle factors and 12 *z*‐scored age gaps, adjusting for chronological age and sex using linear regression (Figure 3A, Table S3).

Smoking‐related behaviors emerged as the most significant risk factors, accelerating aging in most organs, with the most substantial effects observed in the lung, followed by the brain and intestine. For instance, individuals with higher pack years of adult smoking as a proportion of life span exposed to smoking had significantly higher age deviations in the lung, kidney, and intestine. Having ever smoked or currently smoking was linked to accelerated aging, and initiating smoking at a younger age further contributed to this effect. Frequent alcohol consumption showed the largest acceleration of aging in adipose, liver, and kidney, but appeared to slightly decelerate aging in the intestine. Strikingly, drinking alcohol with meals had a protective effect on most organs. More days per week of moderate and vigorous physical activity for more than 10 min correlated with decelerated proteomic age, whereas sedentary behaviors, such as television watching and computer use, were linked to accelerated aging. A nutritious diet that included fruits, vegetables, and oily fish was significantly associated with decelerated proteomic aging in adipose, intestine, kidney, and pancreas. However, variation in diet corresponded to accelerated adipose and intestinal age. Poor sleep patterns, including insomnia, daytime napping, and narcolepsy, were also linked to accelerated proteomic aging in some organs, such as the brain and immune system. Higher socioeconomic status, such as a lower Townsend deprivation index, more vehicles in the household, and higher average total household income, was significantly associated with decelerated proteomic aging across most organs.

We also investigated the impact of the diagnosis of chronic diseases before baseline on proteomic aging. Individuals with chronic diseases were grouped into 16 disease categories: Parkinsonism, multiple sclerosis, stroke, dementia, depression, bipolar disorder, schizophrenia, IHD, hypertensive diseases, COPD, CKD, diabetes, cirrhosis, osteoarthritis, osteoporosis, and cancer. On average, individuals with chronic diseases exhibited accelerated aging across most organs relative to their chronological age, typically ranging from −0.17 to 2.85 years (Figures 3B, S6, S7, and Table S5). Individuals with CKD suffered a substantially greater impact, showing age acceleration exceeding 1.5 years in most organs, followed by cirrhosis and diabetes. Organ‐specific ages exhibit substantial heterogeneity across different diseases and even within the disease. Organs directly affected by the underlying disease tended to show the most pronounced age gaps. For example, significant age acceleration was observed in the kidney in CKD (+2.85 years), the pancreas in diabetes (+0.86 years), the brain in depression (+0.42 years), and the heart in IHD (+0.78 years). Notably, some diseases also showed accelerated aging in organs not typically associated with their primary pathology. Accelerated brain aging was observed not only in neurological disorders such as multiple sclerosis (+0.85 years), stroke (+0.72 years), and schizophrenia (+0.50 years) but also in CKD (+1.78 years), diabetes (+0.71 years), and IHD (+0.33 years). Notably, CKD demonstrated a substantially greater impact, showing age acceleration exceeding 1.5 years in seven organs. In contrast, certain organs exhibited slight proteomic age deceleration in specific diseases, although these findings were not statistically significant (Figure S7).

### 3.5 Proteomic Age Gap Predicts Mortality

**FIGURE 4:**
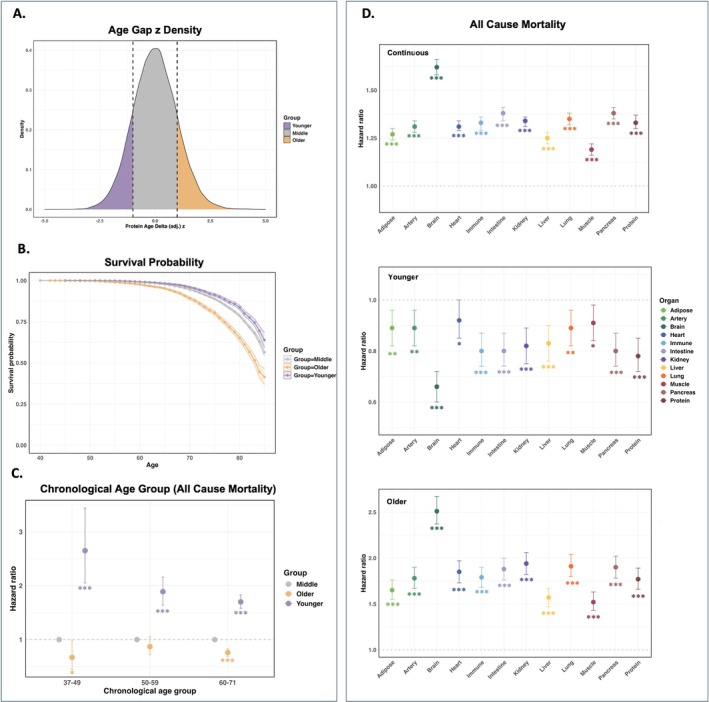
Associations between organ‐specific age gaps and all‐cause mortality. (A) Distribution of *z*‐scored “Protein” age gaps across individuals. (B) Survival probability by chronological age, stratified by “Protein” age gap groups. (C) HR for all‐cause mortality across different chronological age groups. (D) Associations between *z*‐scored organ‐specific age gaps and all‐cause mortality. Significance levels are denoted as follows: “***” indicates *p* < 0.001, “**” indicates 0.001 ≤ *p* < 0.01, ‘*’ indicates 0.01 ≤ *p* < 0.05, and no asterisk indicates *p* ≥ 0.05. “Continuous” refers to treating the *z*‐scored age gap as a continuous variable, while “Younger” and “Older” represent the extreme age groups, with the “Middle” group serving as the reference group.

We separated individuals into three groups (Younger: *z*‐scored age gap < −1; Older: *z*‐scored age gap > 1; Middle: *z*‐scored age gap between −1 and 1) based on the age gap (Figure 4A). The organ‐specific age gap was strongly associated with all‐cause mortality (Figures 4B and S8). To evaluate these relationships, we conducted a prospective analysis using multivariable Cox proportional hazards models, adjusting for chronological age and sex. In the “Protein” model, each unit increase in the *z*‐scored age gap was linked to a higher risk of all‐cause mortality, with a hazard ratio (HR) of 1.33 (95% CI: 1.30 to 1.37, *p* < 0.001). Further analysis stratified by chronological age revealed that a higher “Protein” age gap was associated with increased mortality risk across all age groups (Figure 4C). Notably, among individuals with the same proteomic age gap, chronologically younger people faced a higher risk for all‐cause mortality. This suggests that accelerated proteomic aging occurring in early life is particularly detrimental, leading to a disproportionately greater risk of death.

Regarding the association between organ‐specific age gaps and all‐cause mortality, an increased organ age gap was consistently linked to a higher mortality risk. The strongest association was observed for brain age (HR = 1.62, 95% CI: 1.58 to 1.66, *p* < 0.001), while the weakest was found for muscle age (HR = 1.19, 95% CI: 1.16 to 1.22, *p* < 0.001) (Figure 4D). Comparing individuals in the “Older” group versus those in the “Middle” group, those with an accelerated aging process demonstrated a higher risk of all‐cause mortality. In contrast, individuals in the “Younger” group showed a markedly reduced mortality risk across most organ systems, except for muscle (HR = 1.10, 95% CI: 1.02 to 1.18, *p* = 0.018). A significant non‐linear relationship between organ‐specific proteomic age and all‐cause mortality was observed, indicating that accelerated proteomic age was significantly associated with a higher risk of all‐cause mortality, while decelerated proteomic age was associated with a lower risk of all‐cause mortality (Figure S9).

### 3.6 Organ‐Specific Age Predicts Future Diseases

**FIGURE 5:**
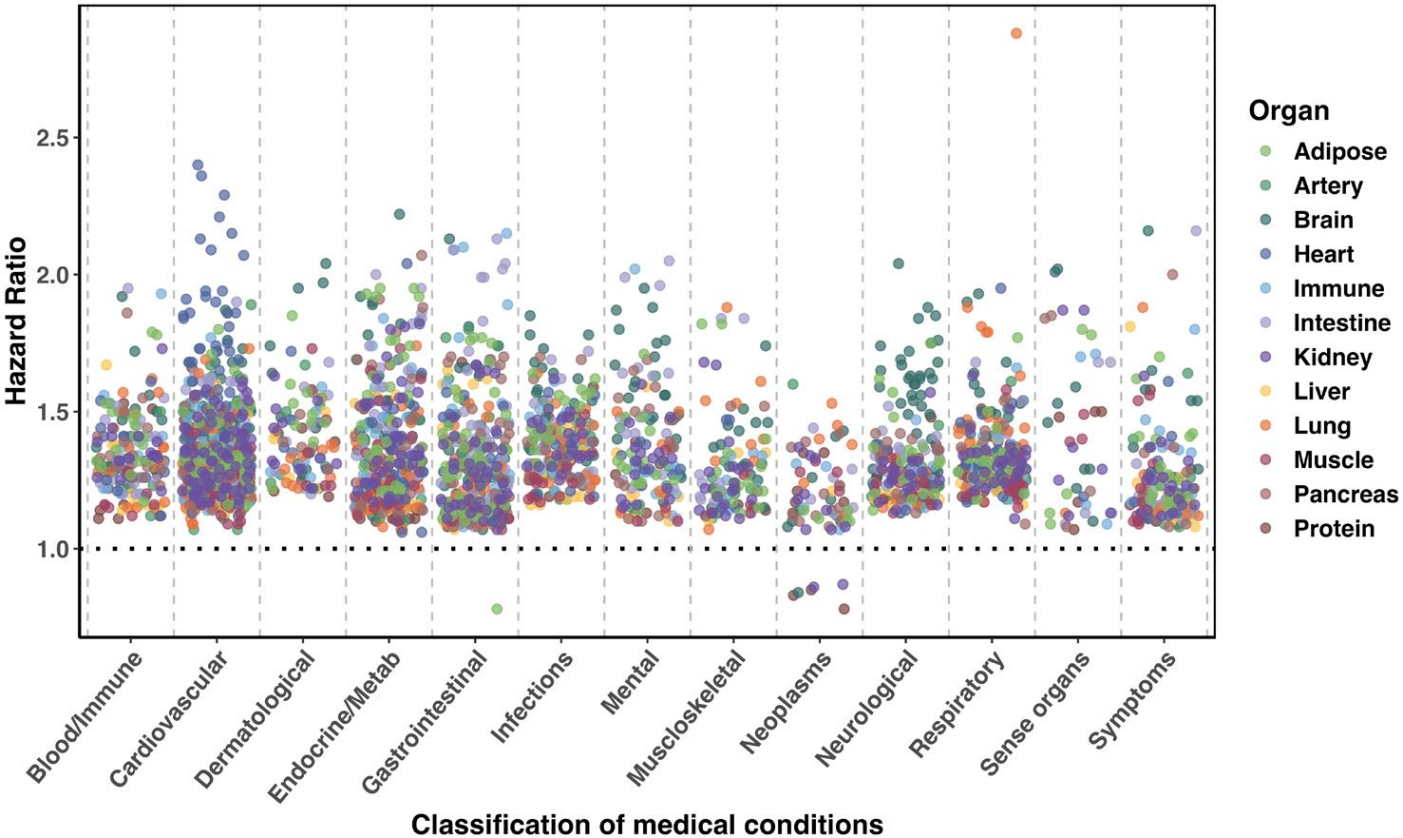
Associations between the *z*‐scored age gap and the risk of developing various medical diseases. Bonferroni correction was used to control the family‐wise error rate (FWER) across 12 × 657 = 7884 tests. The color of the data point indicates the specific aging clocks. All associations were tested by multivariable Cox proportional hazards models and were adjusted for chronological age and sex. Only significant associations (*p* < 0.05/7884) are shown in the figure.

We examined whether the “Protein” age gap could predict future health outcomes and disease risk. Using multivariable Cox proportional hazards models and adjusting for chronological age and sex, we assessed associations across 657 diseases with at least 100 incident cases. The acceleration in the “Protein” aging was significantly associated (202 significant associations, [30.7% of all diseases tested]) with an elevated risk (HR range: 1.07 to 1.68, [100% of significant associations]) of some diseases (Figure 5, Table S6). The strongest associations were observed in cardiovascular, endocrine/metabolic, neurological, and respiratory diseases, indicating that accelerated proteomic aging, as reflected by proteins, may be an early predictor of systemic health decline. For instance, individuals with accelerated “Protein” aging exhibited an increased likelihood of developing diseases such as hepatic failure (HR = 1.68, 95% CI: 1.43 to 1.98, *p* < 0.001), ventricular tachycardia (HR = 1.66, 95% CI: 1.46 to 1.89, *p* < 0.001), hypoglycemia (HR = 1.54, 95% CI: 1.39 to 1.70, *p* < 0.001), and vascular dementia (HR = 1.48, 95% CI: 1.30 to 1.68, *p* < 0.001).

We also examined the relationship between *z*‐scored organ‐specific age gaps and future disease risk (Figures 5 and S10, Table S6). The strongest associations were observed in cardiovascular, endocrine/metabolic, infections, neurological, and respiratory diseases. A consistent pattern was observed where accelerated aging of a specific organ was linked to an increased risk of diseases primarily affecting that organ system. For instance, accelerated aging of the artery and heart was significantly correlated with greater susceptibility to cardiovascular diseases, and accelerated brain aging was significantly associated with an elevated risk of mental health and neurological disorders. Interestingly, accelerated aging in one organ could also be associated with increased risk of diseases beyond its primary pathological domain. One prominent example was the significant association between accelerated kidney aging and an elevated risk across multiple disease systems, including cardiovascular, endocrine/metabolic, neurological, and respiratory diseases. Interestingly, accelerated adipose aging was linked to a reduced risk of inguinal hernia (HR = 0.78, 95% CI: 0.74 to 0.83, *p* < 0.001).

### 3.7 Higher Organ‐Specific Age Increases Age‐Related Chronic Disease Risk

**FIGURE 6:**
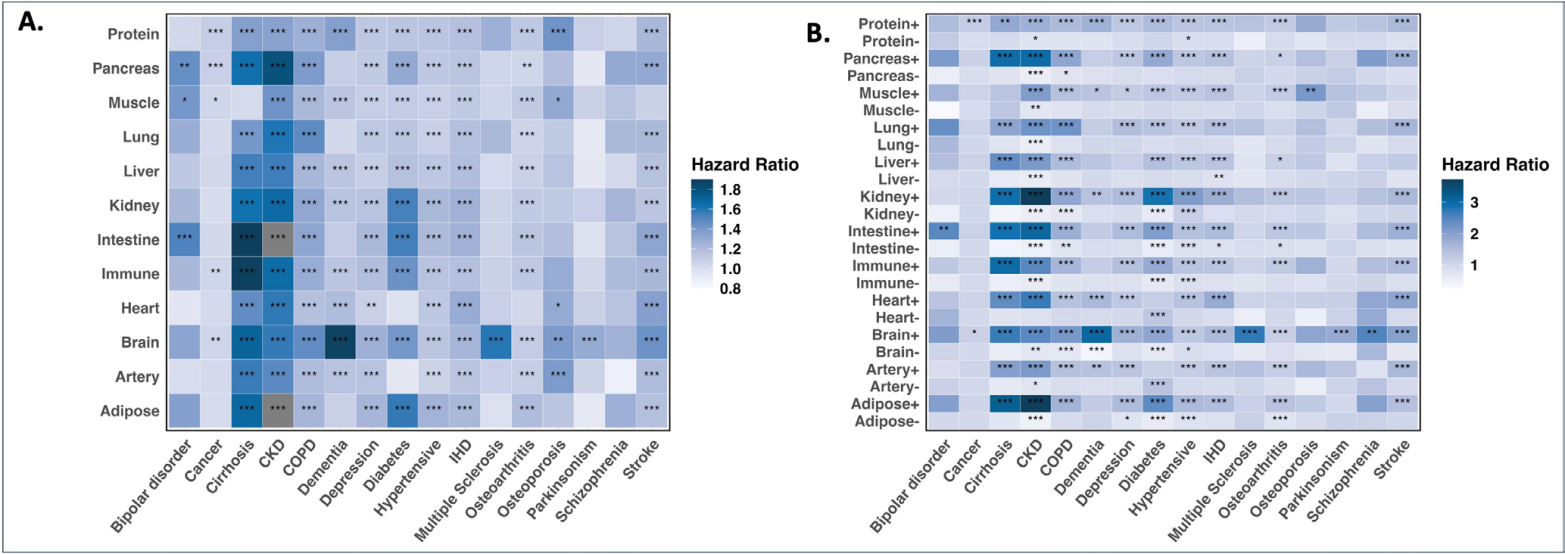
Associations between organ‐specific aging and chronic disease risk. (A) HRs for the associations between *z*‐scored age gaps and 16 age‐related chronic diseases. Bonferroni correction was used to control the family‐wise error rate (FWER) across 12 × 16 = 192 tests. (B) HRs for the associations between extreme organ agers and chronic disease risk. Bonferroni correction was used to control the family‐wise error rate (FWER) across 12 × 16 × 2 = 384 tests. Cox proportional hazards regression was used to determine the association between organ‐specific aging and chronic disease risk, adjusting for chronological age and sex. Heatmaps colored by HR are shown in the figure. “+” indicates organ aging was in the “Older” group, while “‐” indicates organ aging was in the “Younger” group. “***” indicates *p.correct* < 0.001, “**” indicates 0.001 ≤ *p.correct* < 0.01, “*” indicates 0.01 ≤ *p.correct* < 0.05, and no asterisk indicates *p.correct* ≥ 0.05.

To further explore the relationship between organ age and future chronic disease risk, we examined the associations between 12 proteomic aging clocks and 16 major chronic diseases (Figure 6A, Table S7). The results revealed significant correlations between increased organ age and elevated risk for multiple chronic diseases. Specific organs, such as the heart, liver, and immune system, exhibited particularly strong associations with disease susceptibility, suggesting that some organs may be more vulnerable to the effects of aging. For example, an increase in kidney age was strongly associated with CKD (HR = 1.67, 95% CI: 1.63 to 1.71, *p* < 0.001), diabetes (HR = 1.54, 95% CI: 1.51 to 1.58, *p* < 0.001), and several other chronic diseases. Notably, predicted brain age showed significant associations with the risk of most major chronic diseases, underscoring its potential as a broad indicator of systemic aging and disease vulnerability.

We further examined how extreme deviations in organ age influenced disease risk (Figure 6B, Table S8). A positive extreme organ age indicates that the predicted organ age is in the “Older” group. In contrast, a negative extreme organ age suggests that the predicted organ age is in the “Younger” group. Individuals in the “Older” group of specific organ age faced a significantly increased risk of developing chronic diseases. In contrast, the “Younger” group exhibited a lower risk, suggesting that maintaining a decelerated proteomic age in specific organs may confer protective health benefits. For instance, an “Older” kidney ager was associated with an increased risk of cirrhosis (HR = 2.90, 95% CI: 2.27 to 3.69, *p* < 0.001) and CKD (HR = 3.70, 95% CI: 3.39 to 4.03, *p* < 0.001). Conversely, “Younger” kidney ager had a reduced risk of diabetes (HR = 0.62, 95% CI: 0.54 to 0.71, *p* < 0.001), CKD (HR = 0.55, 95% CI: 0.46 to 0.65, *p* < 0.001), and other diseases.

## 4 Discussion

Our analysis of the UKB cohort offers a comprehensive assessment of the associations between estimated proteomic age, lifestyle factors, and diseases, highlighting the role of organ‐specific proteomic aging in shaping health and disease trajectories. The findings demonstrate that proteomic age gaps effectively capture distinct organ‐specific aging patterns while highlighting shared mechanisms across organ systems. These aging dynamics are modulated by lifestyle behaviors and existing disease burdens, which in turn influence mortality rates and the risk of developing future diseases. This underscores the importance of systemic interactions and potentially modifiable biological pathways in shaping the overall aging process, advancing our understanding of how proteomic signatures can inform precision prevention strategies.

Our study reveals sex‐based differences in proteomic age gaps, with females generally exhibiting narrower distributions than males (Antal et al. 2025; Shen et al. 2024; Tian et al. 2023). This suggests accelerated aging in males, potentially due to behavioral factors, such as elevated smoking and alcohol consumption rates among males (Im et al. 2023; John et al. 2021), or inherent biological variations, including slower inflammation resolution, faster telomere shortening, and divergent immune responses (Goeminne et al. 2025; Klein and Flanagan 2016; Rathod et al. 2023). To capture these sex‐specific dynamics more precisely, this study employed sex‐stratified elastic net models using Olink proteins from the UKB, outperforming covariate adjustments in revealing dimorphic protein effects. In prior proteomic studies, sex was entered as a covariate when training aging models (Oh et al. 2023); despite this methodological difference, the top‐ranked proteins based on the absolute coefficient estimates from our sex‐stratified analyses showed striking agreement with theirs, underscoring the robustness of the key biological signals across both approaches.

Our findings link accelerated proteomic aging to heightened risks of organ‐specific diseases and overall mortality. For example, accelerated kidney aging associates with diabetes and hypertension, heart aging with cardiac events, and brain aging with neurological disorders, mirroring patterns reported by Oh et al. (2023). Building on these observations, our study incorporates an expanded set of 86 lifestyle factors, revealing modifiable factors such as smoking, which hasten aging in the lung and kidney. Moreover, the PheWAS encompassing 657 diseases provides a broader understanding of systemic interconnections. Both studies highlight increased mortality hazards linked to accelerated aging, with particularly pronounced effects for brain aging and among chronologically younger individuals (Oh et al. 2025). Variations in effect magnitudes between studies may arise from differences in cohort demographics, sample sizes, or proteomic platforms, emphasizing the need for cautious interpretation across contexts.

We further explored the influence of modifiable factors on proteomic aging, focusing on behaviors such as smoking and alcohol consumption. Consistent with previous studies, our findings suggest that environmental and behavioral factors significantly influence proteomic aging (Burtscher et al. 2022; D'Angelo 2023; Di Giosia et al. 2022; Wang et al. 2022). Smoking, for instance, accelerates aging in the lung, kidney, and intestine, probably due to the cumulative impact of inflammatory and oxidative damage in tissues directly exposed to inhaled or metabolically processed toxins (King et al. 2017; Kivimäki et al. 2025). However, exposures do not always yield uniform outcomes. Alcohol intake during meals, while accelerating aging in some organs, correlates with deceleration in others (Åberg et al. 2023; Shield et al. 2013). These inconsistent effects may reflect differences in metabolic timing, modulation of gut microbiota, or organ‐specific regulation of inflammatory responses (Betrapally et al. 2016; Lowe et al. 2018).

To better understand the mechanisms behind accelerated organ aging in the presence of chronic diseases, it is essential to consider several interconnected biological processes that contribute to this phenomenon. The clustering of accelerated aging across multiple organs in individuals with chronic diseases suggests shared mechanisms, including chronic low‐grade inflammation, mitochondrial inefficiency, oxidative stress, and impaired tissue, which drive systemic aging (Li et al. 2023; Liao and Kennedy 2016). Among these, CKD showed the greatest acceleration effect due to factors like uremic toxins accumulation, low‐grade inflammation, and metabolic disturbances (Faucher et al. 2023; Kooman et al. 2017; Stocker et al. 2023). In alignment with earlier studies, accelerated organ aging elevates hazard ratios for all‐cause mortality, with brain age showing the strongest association. This is associated with global physiological stress, functional decline, and neurodegenerative changes (Antal et al. 2025; Cole et al. 2018). Notably, accelerated biological age in chronologically younger individuals may reflect deviation from normal aging, possibly driven by early‐life exposures, genetics, or environmental stressors (Argentieri et al. 2025; Colich et al. 2020; Zhao et al. 2024).

Several limitations should be considered when interpreting these findings. First, proteomic aging models were trained and tested on a single UKB cohort, which may introduce overfitting concerns and affect the generalizability of the results. Performance metrics like MAEs or correlations between predicted and chronological age may be optimistically biased. External validation on diverse datasets is thus essential to substantiate the models' predictive power and mitigate these risks. Second, although the proteomic aging models were trained on a large population, they may still be affected by residual confounding or unmeasured variables. Third, the age gaps were derived from a single time point in a cohort aged between 39 and 71 and predominantly of European ancestry, limiting insights into longitudinal changes, broader age groups, or ethnic diversity. Fourth, plasma protein may only partially capture organ‐specific biological processes, as circulating protein levels may not fully reflect tissue‐level alterations or localized molecular events. Fifth, technical factors such as measurement variability, platform‐specific biases, and batch effects could affect the accuracy and generalizability of the age predictions. Lastly, while our findings link proteomic age gaps to several lifestyle, environmental, and disease variables, the underlying biological mechanisms remain complex and incompletely understood. Future studies incorporating longitudinal measurements, diverse populations, interventional data, and multi‐omics integration will be essential to clarify the regulatory basis of proteomic aging and its potential clinical relevance.

In summary, this large‐scale analysis advances the field by identifying modifiable drivers and risks, positioning proteomic aging as a tool for precision prevention. Organ aging is dynamic and modifiable, shaped by baseline health conditions and environmental exposures. Accelerated organ aging is associated with a higher incidence of chronic diseases and an increased risk of all‐cause mortality, particularly when it begins earlier in life. These findings enhance our understanding of proteomic aging patterns across multiple organ systems and provide a foundation for developing targeted strategies to reduce disease risk and promote healthy longevity.

## Author Contributions

Conceptualization: M.P.S., L.L. Methodology: Q.W., Q.H., M.P.S., L.L. Investigation: Q.W., J.H., M.P.S., L.L. Visualization: Q.W., J.H. Supervision: M.P.S., L.L. Writing – original draft: Q.W., J.H. Writing – review and editing: Q.W., J.H., Q.H., M.S., M.P.S., L.L.

## Conflicts of Interest

M.P.S. is a cofounder and scientific advisor of Personalis, SensOmics, Qbio, January AI, Fodsel, Filtricine, Protos Biologics, RTHM, Iollo, Marble Therapeutics, Crosshair Therapeutics, Exposomics, InVu health, Next Thought AI, Orange Street Ventures, RTHM, and Mirvie. M.P.S. is a scientific advisor of Jupiter Therapeutics, Neuvivo, Abbratech, Applied Cognition, Enovone, M3 Helium, Onza, Sigil Bioscience, TranscribeGlass, WndrHLTH, and Mitrix. M.P.S. is an investor and scientific advisor of R42 and Swaza. M.P.S. is an investor in Repair Biotechnologies. All other authors declare that they have no competing interests.

## Supporting information


**Supplementary Figure 1.** Number of organ‐specific proteins used for proteomic age estimate in each organ.
**Supplementary Figure 2.** Coefficient estimates in sex‐stratified elastic net regularization models for top‐ranked proteins.
**Supplementary Figure 3.** Mean absolute error of the training and test sets for different models.
**Supplementary Figure 4.** Scatter plot of the prediction age derived from sex‐stratified models and both‐sexes models.
**Supplementary Figure 5.** Aging model prediction and bias correction for 11 organ systems.
**Supplementary Figure 6.** Age gap density plot of individuals with specific chronic disease.
**Supplementary Figure 7.** Mean *z*‐scored age gaps for individuals affected by 16 chronic diseases.
**Supplementary Figure 8.** Survival probability by chronological age, stratified by age gap groups.
**Supplementary Figure 9.** Log(HRs) and 95% CIs from Cox proportional hazards models for all‐cause mortality.
**Supplementary Figure 10.** Association between *z*‐scored age gap and future disease risk.

**Table S1:** Coefficient estimates in proteomic aging models for females.
**Table S2:** Coefficient estimates in proteomic aging models for males.
**Table S3:** Associations between lifestyle/environmental factors and organ aging.
**Table S4:** ICD codes of 16 chronic diseases.
**Table S5:** Mean *z*‐scored age gap of individuals diagnosed with specific chronic disease.
**Table S6:** PheWAS results of *z*‐scored organ‐specific proteomic age gap and incident disease.
**Table S7:** Associations between organ‐specific aging and 16 chronic diseases.
**Table S8:** Associations between organ‐specific aging and 16 chronic diseases in extreme agers.

## Data Availability

The data used in this study, sourced from the UK Biobank, are publicly available to registered researchers through the UK Biobank data access protocol. Information regarding registration and access to study materials is available online at www.ukbiobank.ac.uk. All other data needed to evaluate the conclusions in the paper are available in the main text or the Supporting Information. Data processing, analysis, and presentation were conducted using publicly available R libraries: data.table (v1.16.0), dplyr (v1.1.4), glmnet (v4.1.8), caret (v6.0.94), impute (v1.80.0), survival (v3.6.4), corrplot (v0.95), cmprsk (v2.1.12), plotRCS (v0.1.5), ggplot2 (v3.5.1), ggpubr (v0.6.0).
