# Supplementary material for: Patterns of Organ‐Specific Proteomic Aging in Relation to Lifestyle, Diseases, and Mortality
Source: Aging Cell. 2025 Oct 8;24(12):e70251. doi: 10.1111/acel.70251 (PMC12686593; doi:10.1111/acel.70251)
Supplement: Supplementary file 1 — Supplementary Figure 1. Number of organ‐specific proteins used for proteomic age estimate in each organ. Supplementary Figure 2. Coefficient estimates in sex‐stratified elastic net regularization models for top‐ranked proteins. Supplementary Figure 3. Mean absolute error of the training and test sets for different models. Supplementary Figure 4. Scatter plot of the prediction age derived from sex‐stratified models and both‐sexes models. Supplementary Figure 5. Aging model prediction and bias correction for 11 organ systems. Supplementary Figure 6. Age gap density plot of individuals with specific chronic disease. Supplementary Figure 7. Mean z‐scored age gaps for individuals affected by 16 chronic diseases. Supplementary Figure 8. Survival probability by chronological age, stratified by age gap groups. Supplementary Figure 9. Log(HRs) and 95% CIs from Cox proportional hazards models for all‐cause mortality. Supplementary Figure 10. Association between z‐scored age gap and future disease risk. [file ACEL-24-e70251-s001.doc]

## **Supplementary Information**

## **1 Supplementary Figures**

**Supplementary Figure 1.** Number of organ-specific proteins used for proteomic age estimate in each organ.

**Supplementary Figure 2.** Coefficient estimates in sex-stratified elastic net regularization models for top-ranked proteins.

**Supplementary Figure 3.** The mean absolute error of the training and test sets for different models.

**Supplementary Figure 4.** Scatter plot of the prediction age derived from sex-stratified models and both-sexes models.

**Supplementary Figure 5.** Aging model prediction and bias correction for 11 organ systems.

**Supplementary Figure 6.** Age gap density plot of individuals with specific chronic disease.

**Supplementary Figure 7.** Mean z-scored age gaps for individuals affected by 16 chronic diseases.

**Supplementary Figure 8.** Survival probability by chronological age, stratified by age gap groups.

**Supplementary Figure 9.** Log(HRs) and 95% CIs from Cox proportional hazards models for all-cause mortality.

**Supplementary Figure 10.** Association between z-scored age gap and future disease risk.

**
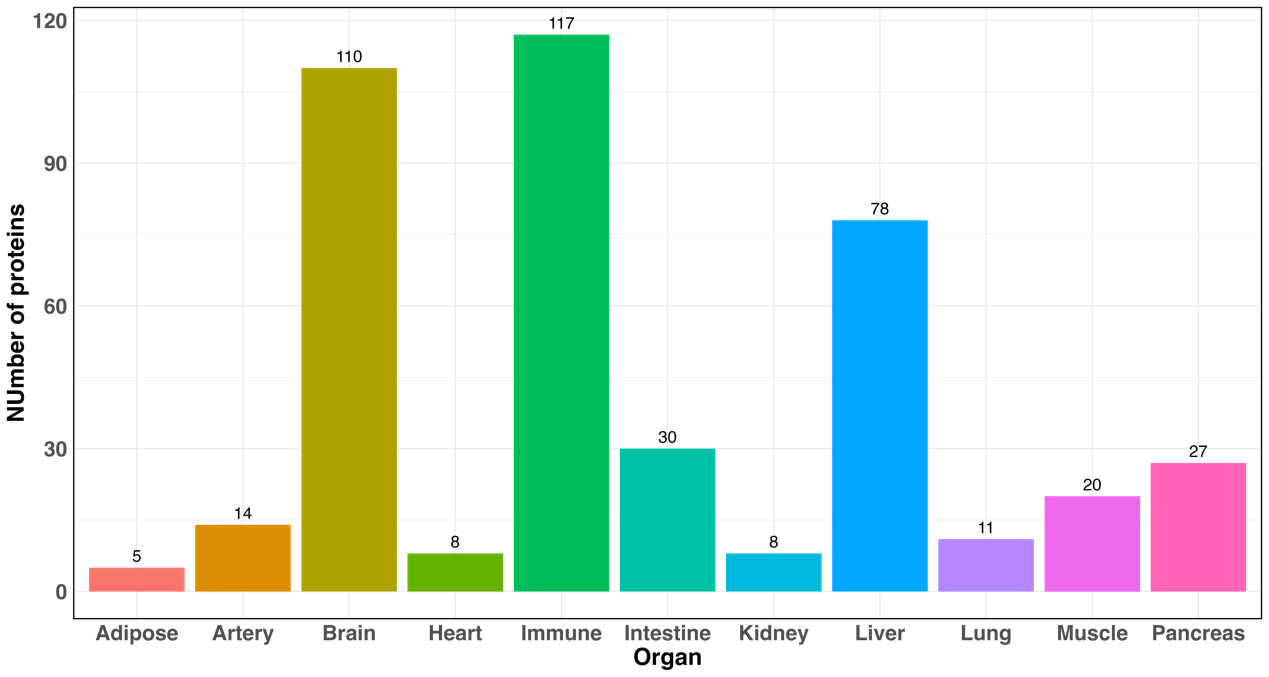
Figure S1. Number of organ-specific proteins used for proteomic age estimate in each organ.**

**
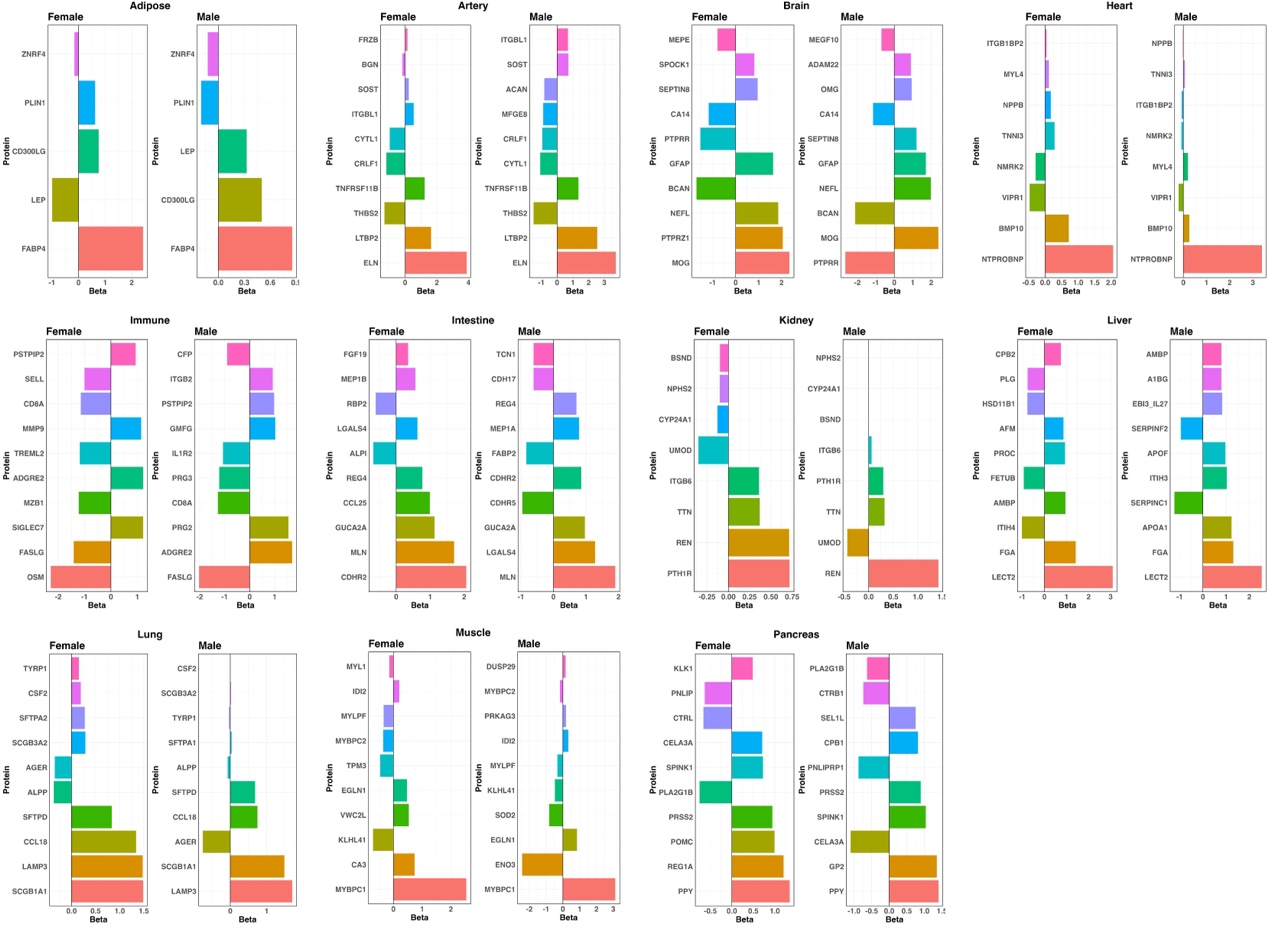
**

**Figure S2. Coefficient estimates in sex-stratified elastic net regularization models for top-ranked proteins.** The top 10 proteins selected in descending order of the absolute values of coefficient estimates for all sex-stratified models across 11 organ systems are shown. If the organ-specific proteins are less than 10, then all proteins are shown in the figure.

**
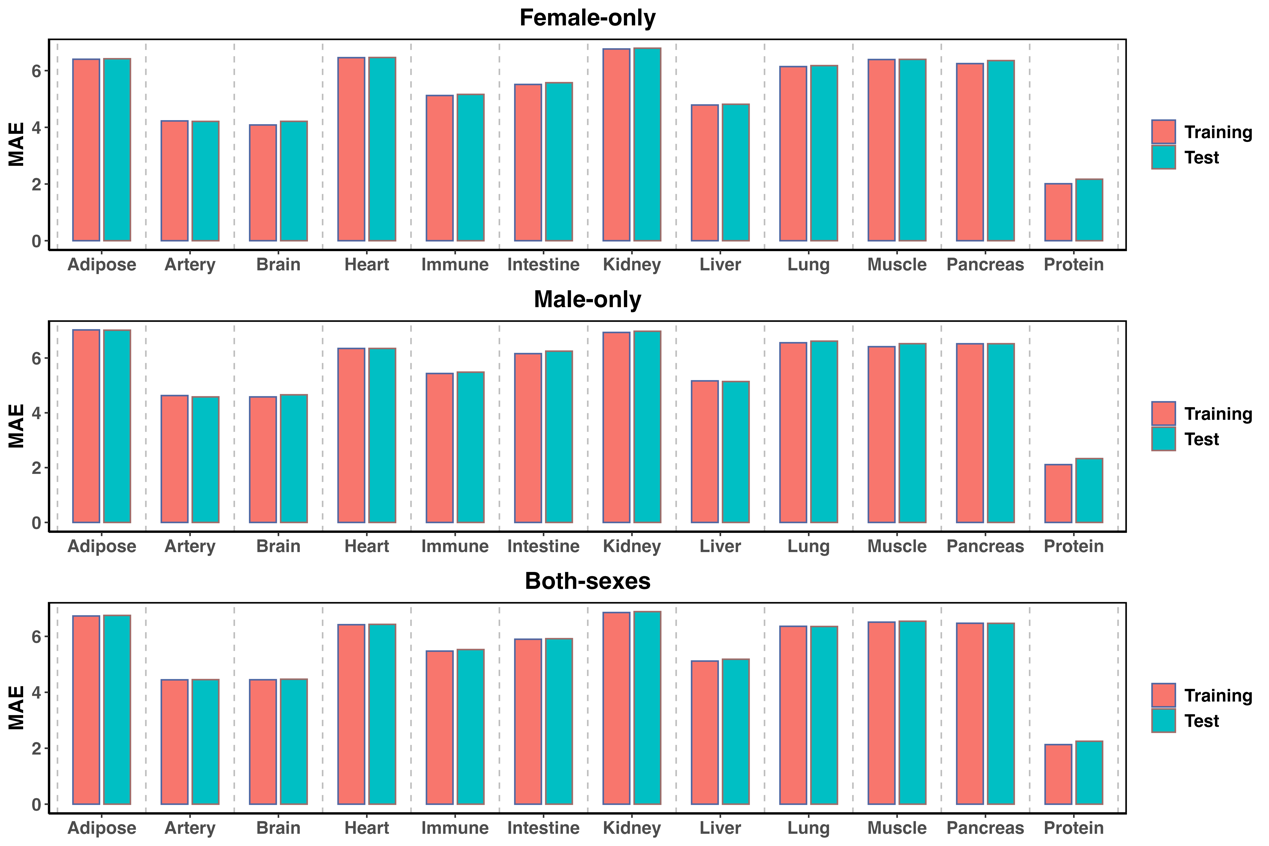
**

**Figure S3. The mean absolute error of the training and test sets for different models**. The mean absolute error of the training and test sets for sex-stratified prediction models and prediction models with sex as a covariate. "Female-only" indicates that models trained and tested in females, "Male-only" indicates that models trained and tested in males, and "Both-sexes" indicates that models trained and tested in all available samples with sex a covariate.

**
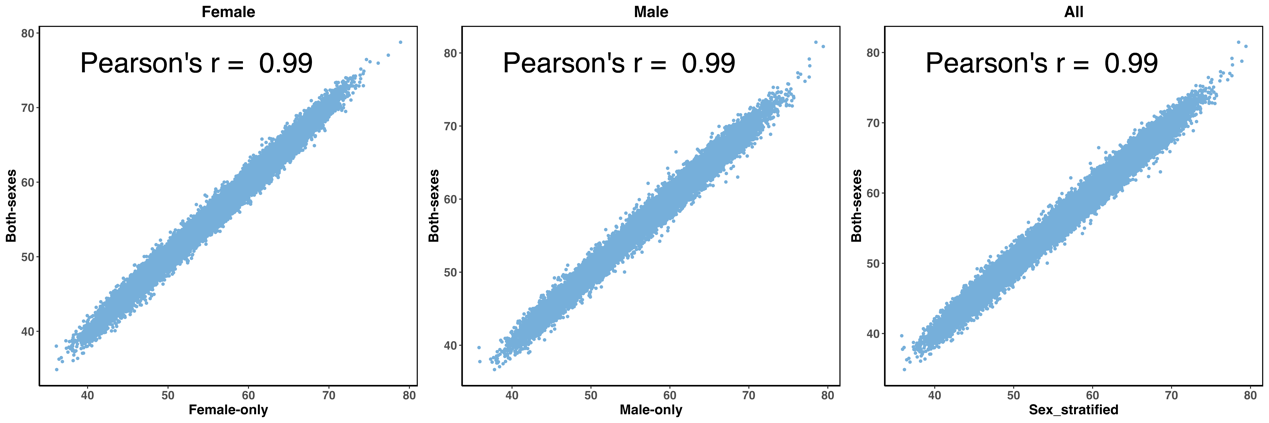
**

**Figure S4. Scatter plot of the prediction age derived from sex-stratified models and both-sexes models**. The prediction age was derived from sex-stratified models and both-sexes models with all proteins in the post-quality control dataset. Pearson’s r was reported in figures.

**
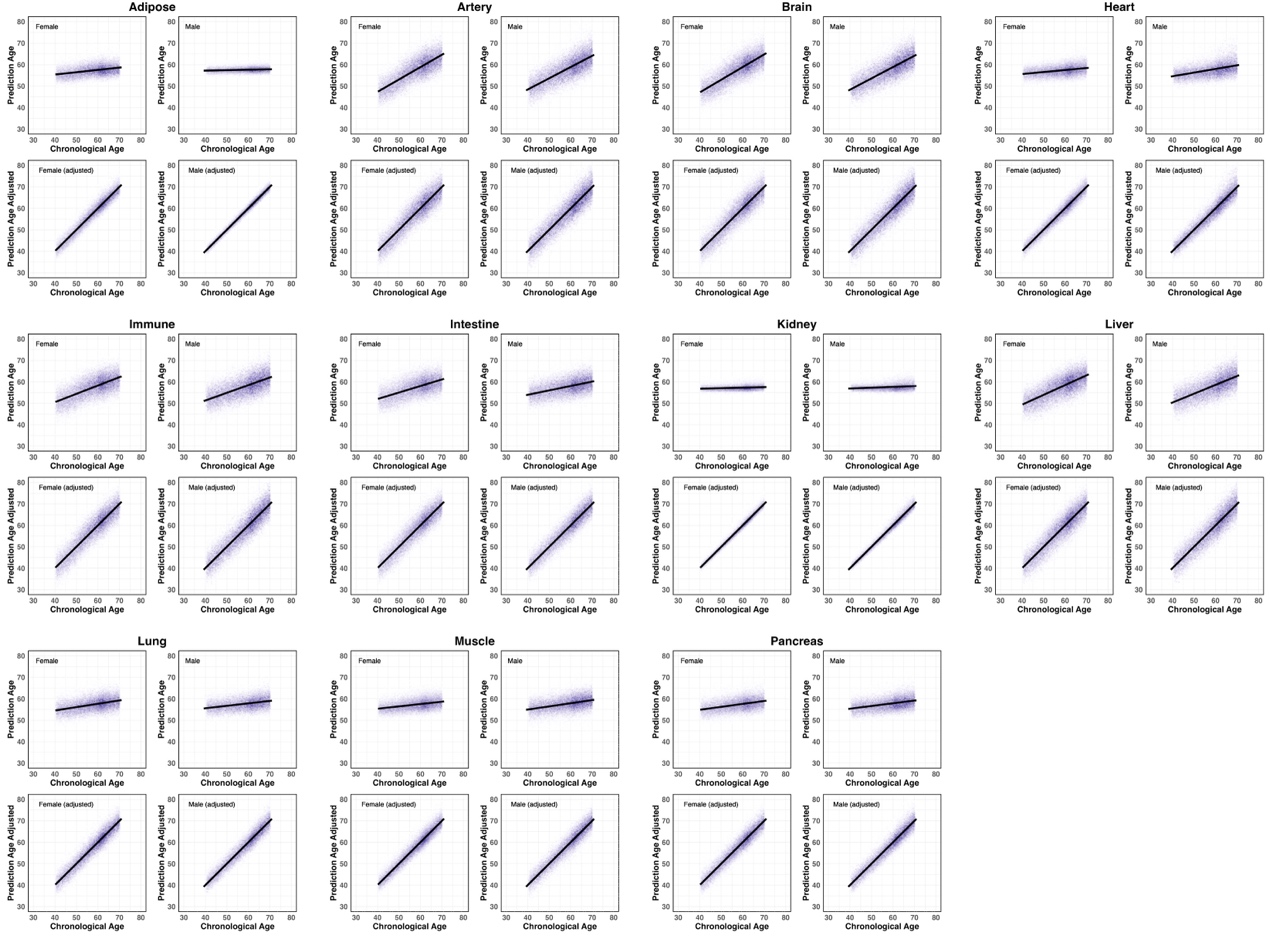
**

**Figure S5. Aging model prediction and bias correction for 11 organ systems.**

**
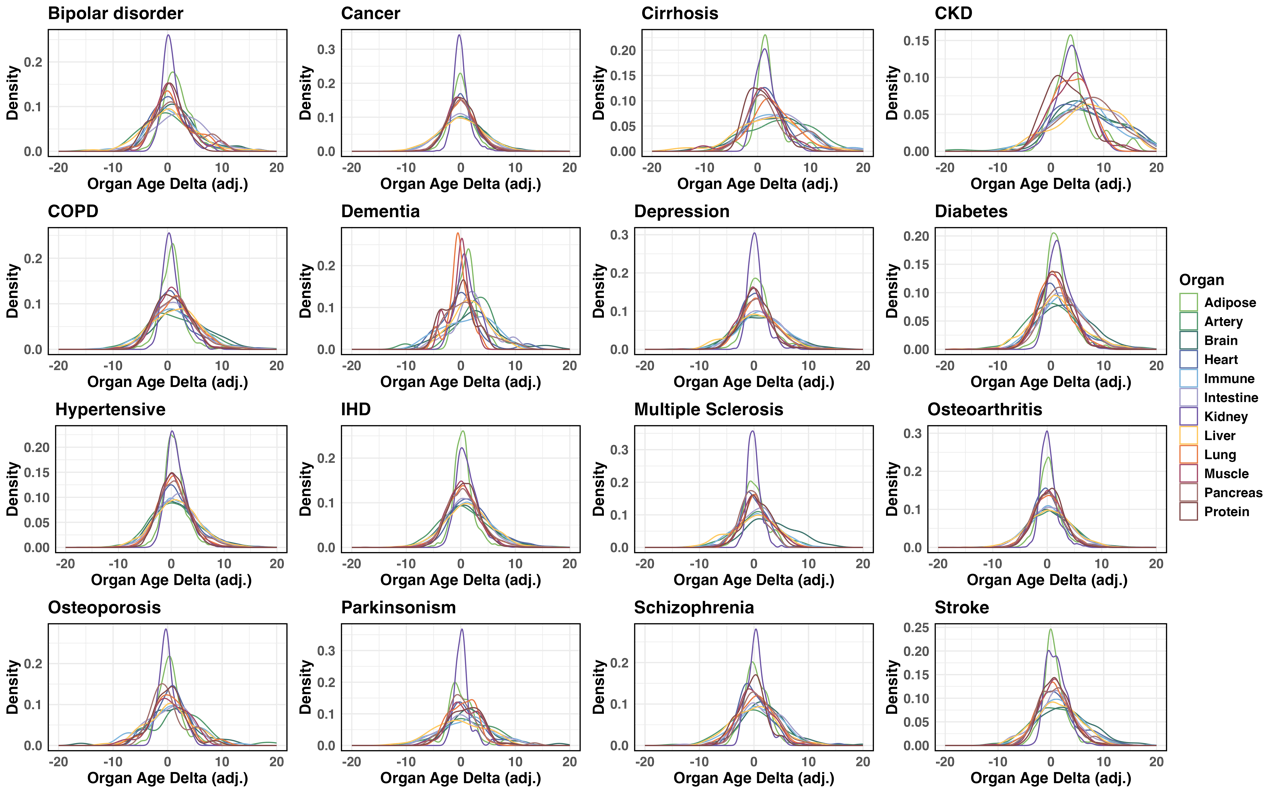
**

**Figure S6. Age gap density plot of individuals with specific chronic disease.**

**
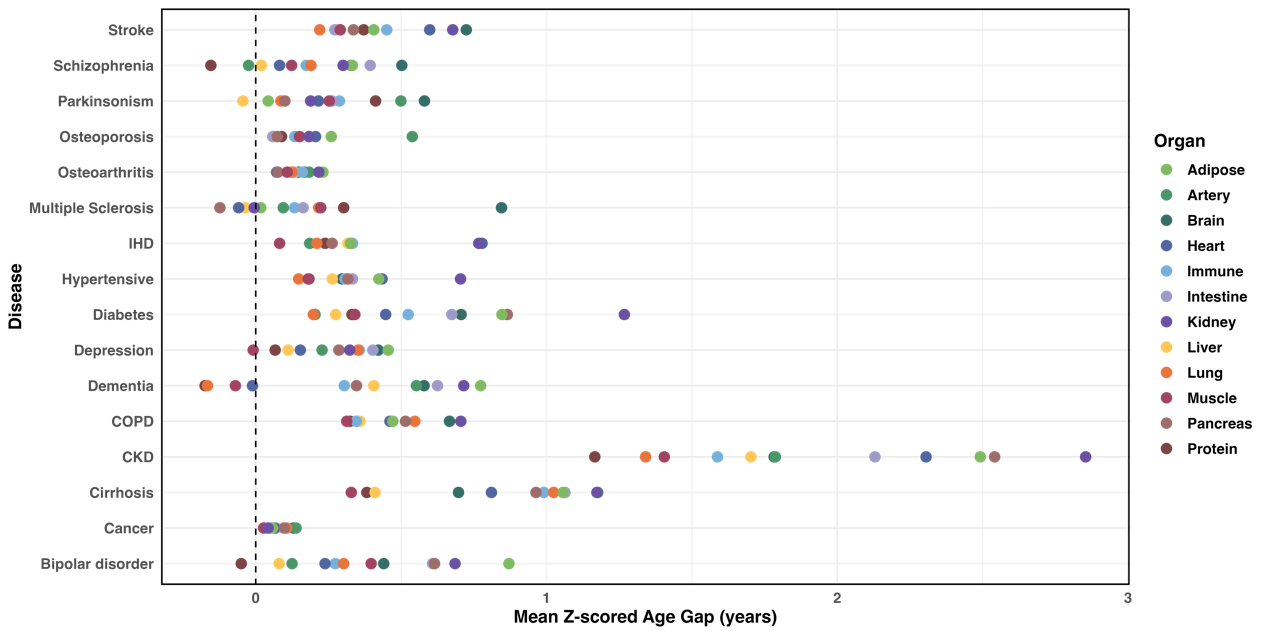
**

**Figure S7. Mean z-scored age gaps for individuals affected by 16 chronic diseases.**

**
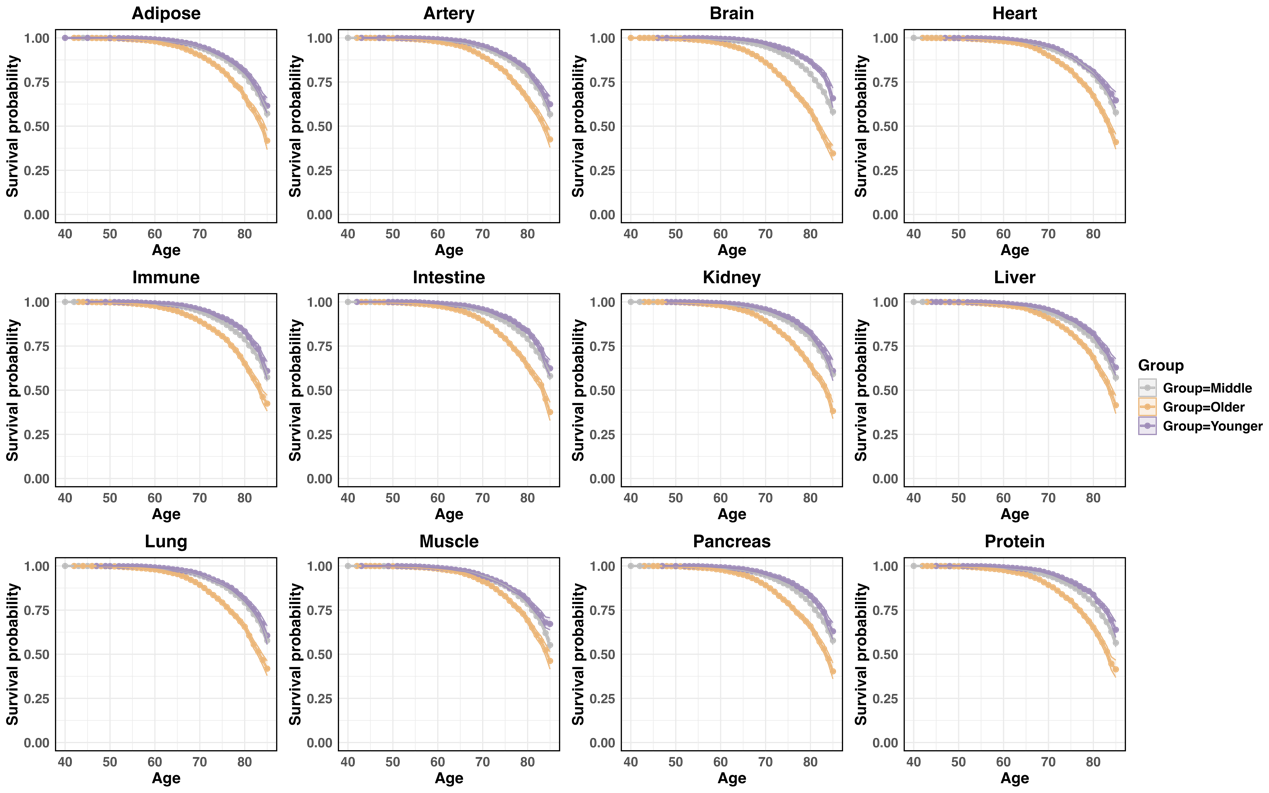
**

**Figure. S8. Survival probability by chronological age, stratified by age gap groups.**

**
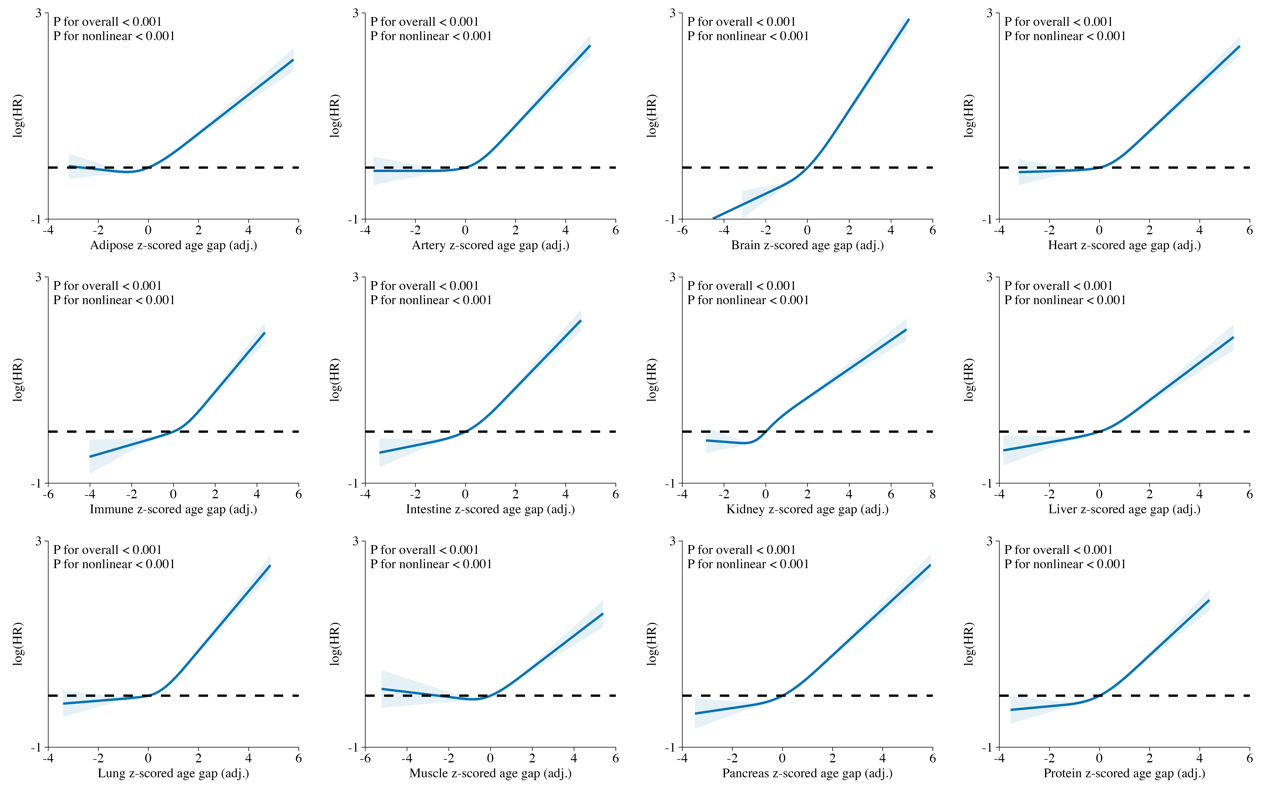
**

**Figure S9. Log(HRs) and 95% CIs from Cox proportional hazards models for all-cause mortality.** A penalized spline function with four degrees of freedom was used. The reference value for interpreting the estimates shown is 0. All models were adjusted for chronological age and sex.


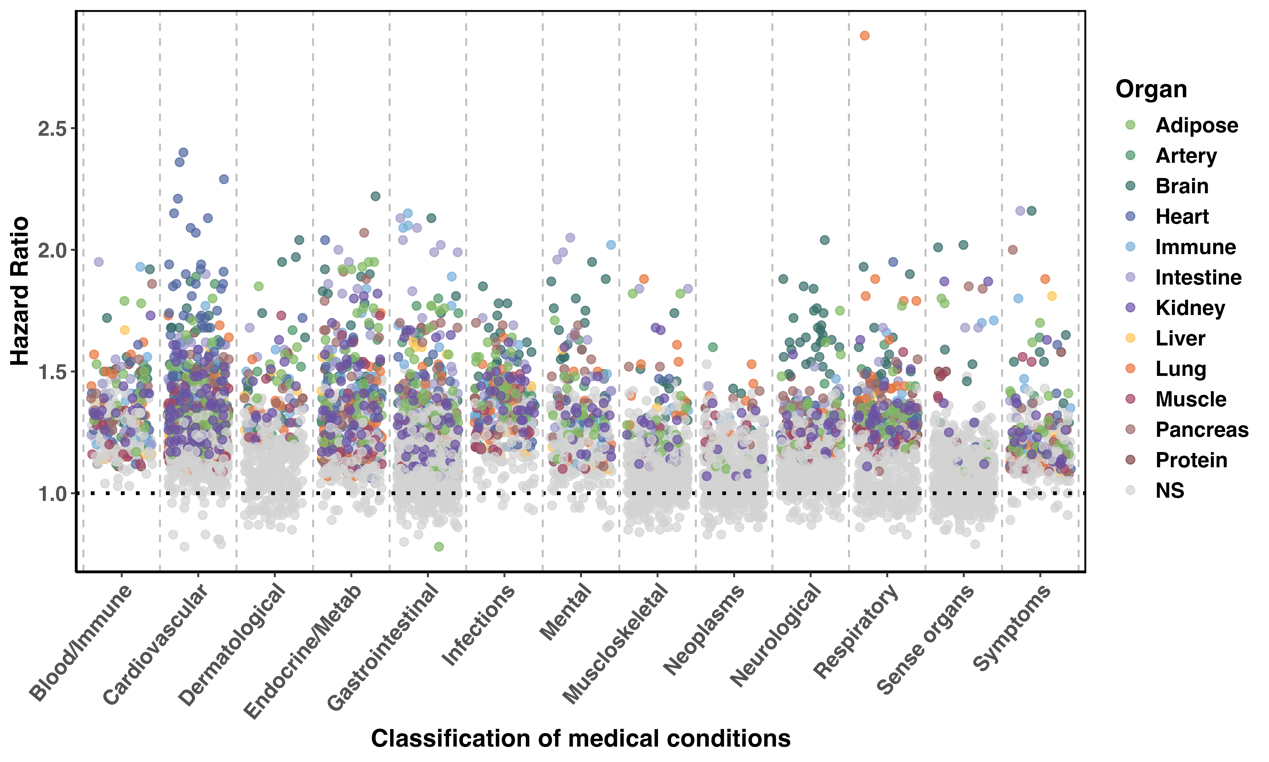


**Figure S10.** **Association between z-scored age gap and future disease risk.** Associations between the z-scored age gap and the risk of developing various medical diseases. Bonferroni correction was used to control the family-wise error rate (FWER) across 12×657 = 7,884 tests. The color of the data point indicates the specific aging clocks. All associations were tested by multivariable Cox proportional hazards models and were adjusted for chronological age and sex.
